# Combining AHP-Entropy Approach with GIS for Construction Waste Landfill Selection—A Case Study of Shenzhen

**DOI:** 10.3390/ijerph15102254

**Published:** 2018-10-15

**Authors:** Zhikun Ding, Menglian Zhu, Zezhou Wu, Yanbin Fu, Xia Liu

**Affiliations:** 1 Department of Construction Management and Real Estate, College of Civil Engineering, Shenzhen University, Shenzhen 518060, China; ddzk@szu.edu.cn; 2 Financial Investment Management Service Center of Honghuagang District, Zunyi 563100, China; mlz321@163.com; 3 Department of Civil Engineering, College of Civil Engineering, Shenzhen University, Shenzhen 518060, China; fuyanbin999@163.com; 4 Architecture Engineering School, Chongqing Vocational Institute of Engineering, Chongqing, 400037, China; liuxiacqu@163.com

**Keywords:** construction waste, landfill selection, AHP-entropy approach, geographic information system (GIS), Shenzhen

## Abstract

With the recent fast economy development and rapid urbanization, the huge generation of construction waste has become a threat to sustainable development in China. Though efforts have been made to promote reuse and recycling of construction waste, landfilling of waste remains the most commonly adapted approach for construction waste disposal. As the space for landfills is limited and because of the negative issues in terms of environmental and social aspects that may be caused, the appropriate site selection of landfills is crucial. With this background, this paper aims to establish a framework for facilitating landfill selection for construction waste. To begin with, a total of sixteen factors that may influence landfill site selection were identified from a literature review. Then, based on the combined analytic hierarchy process (AHP) and entropy method, the weights and the final comprehensive scores of the identified factors were calculated. According to the derived results, potential sites for landfills were divided into three levels, namely the most appropriate (0.38%), appropriate (17.58%), and inappropriate (82.04%). The proposed decision-making methods in this paper can provide a valuable reference for the selection of construction waste landfill sites.

## 1. Introduction

The economy in China has experienced a rapid growth in the past two decades. For example, the urbanization rate increased from 45.2% in 2007 to 57.9% in 2017 [1]. Along with the rapid urbanization, the amount of waste generated from new construction and demolition activities also grew at a high rate [2,3,4]. Currently, although the reuse and recycling of construction waste has been promoted by local governments and academia scholars, the most popular waste disposal approach is still landfilling [5,6,7,8]. Landfills occupy land resources and may cause water pollution, harmful gas emission, and have other negative impacts on the environment [9,10,11,12,13], thus selecting an appropriate construction waste landfill location is essential for sustainable urban development.

In literature, there have been several studies focusing on the site selection of waste treatment facilities, as shown in Table 1, where it can be seen that multi-objective modeling was widely used in early studies, such as Giannikos [14], Rakas, et al. [15], etc. In the study published by Şener, et al. [16], the Geographic Information System (GIS) was introduced to integrate with the Multi-Criteria Decision Analysis (MCDA) to identify the appropriate locations for landfills. After that, GIS has almost become an indispensable tool for making decisions of landfill site selections. At the same time, the Analytical Hierarchy Process (AHP) has been commonly used to evaluate the weights of potential affecting factors.

From the literature review, it can be seen that the current studies on landfill site selection mainly focused on municipal solid waste; studies on how to choose an appropriate location for construction waste landfills are very limited. As the composition of construction waste and municipal solid waste is quite different [44], there is a necessity to investigate the specific influencing factors for construction waste landfill selection. In this circumstance, this paper aims to establish an evaluation framework for site selection of construction waste landfills.

In this study, Shenzhen is selected to conduct a case study. The potential influencing factors of site selection for construction waste landfills are identified based on the literature review. Then, an expert scoring process is employed, and the weight of each influencing factor is determined by the analytic hierarchy process (AHP) and entropy method. Furthermore, the geographic information system (GIS) technique is used for spatial data screening and calculation. Finally, an evaluation framework for site selection of construction waste landfills is established. This model can be used to analyze the impacts of environmental as well as social and economic factors on site selection of construction waste landfills.

## 2. Materials and Method

### 2.1. The Study Area

In this research, the selected area for the case study is Shenzhen. Shenzhen is in the south of Guangdong Province with a total area of 1996.85 km^2^, including eight major administrative districts. In 2017, the gross domestic product (GDP) in Shenzhen reached US$ 338 billion with a GDP growth rate of 8.8%, playing the role as the engine of the Pearl River Delta [45]. It is the first special economic zone established by the Central Government of China.

With the rapid economic development, Shenzhen is facing a problem of land resources limitation. The lands for constructing new buildings are mainly from urban renewal which causes a huge amount of construction waste. According to the Shenzhen Environmental Status Bulletin, the amount of construction waste reached 1.122 million tons, accounting for 16.4% of municipal solid waste. However, there are only five construction waste treatment facilities in Shenzhen and the total capacity is 5.2 million tons which meets the limit. Therefore, how to reasonably deal with the generated construction waste is an important problem to be solved in Shenzhen.

### 2.2. Influencing Factors for Locating Construction Waste Landfills

There are many influencing factors to be considered for the site selection of landfills. For example, El Baba, Kayastha, and De Smedt [30] took a series of environmental factors, such as soil type, depth of groundwater, rainfall, and elevation, into consideration when selecting landfill sites. Social factors and economic factors should also be considered when selecting landfill sites. Wang, Qin, Li, and Chen [23] involved economic factors and political opposition to optimize the location of landfills. Gorsevski, Donevska, Mitrovski, and Frizado [27] tried to reduce the impact on the surrounding residents when determining landfill sites. Eskandari, Homaee, and Mahmodi [26] considered 16 environmental, economic, social, and cultural constraints, such as groundwater depths, airports, historical and cultural sites, earthquake areas, and mines, to create a site selection model. Abd-El Monsef [29] considered other factors, such as urban landfill traffic routes, coasts, high flood risk zones, faults, and cracks, in their study. Rahmat, Niri, Alavi, Goudarzi, Babaei, Baboli, and Hosseinzadeh [33] also identified several parameters, such as distance to groundwater, distance to surface water, ecosystem, land cover, distance to city and countryside, and land use, to establish an evaluation framework for landfill site selection.

Based on the previous research and local regulations/technical standards in Shenzhen, this study identified the potential influencing factors of choosing construction waste landfill sites. These factors can be categorized into three criteria: environmental criteria, social criteria, and economic criteria. Furthermore, some factors can be divided into more detailed sub-criteria, as shown in Table 2.

### 2.3. Research Methodology

The research methodology used in this study includes an AHP-entropy approach which is combined with GIS technology. The AHP-entropy approach is used to determine the weights of each influencing factor, while the GIS technology can be used to calculate and display the results on a geographic figure.

#### 2.3.1. AHP-Entropy Approach

There are many decision-making techniques in literature [46,47,48,49,50], this paper adopts an AHP-entropy approach. The AHP-entropy approach is a combination of AHP and entropy method. AHP is an effective multi-criteria decision-making method to determine weighting coefficients [51,52,53]. It uses mathematical language to build a hierarchical model to describe the decision-making process [54]. To implement the AHP method, a hierarchical structure should be established first. The hierarchical structure can disassemble the problem issue into various components. Then, a judgment matrix should be established and calculated to determine the relative importance of each level according to objective reality judgments. A consistency check is important and essential, and the consistency ratio (CR) should not exceed 0.10. Finally, the weights can be determined based on the results of previous process.

However, AHP may produce problems such as the uncertainty of the scale or the loss of information because it requires less quantitative data [55]. In this circumstance, the entropy method is introduced, integrated with AHP. Entropy is a core concept of information theory, it is an expression of the disorder, or randomness of a system [56]. The entropy method is an objective weighting method and a typical process is as follows: (1) standardize the evaluation matrix; (2) calculate the specific density of the evaluation value of index *C_j_* for object *B_i_*, (3) establish a density matrix; (4) calculate the entropy value and entropy weight of index *C_j_*.

The AHP-entropy approach utilizes the results derived from the AHP and entropy method, a fine-tuned weight value is calculated using Equation (1):

(1) $$w_{j}^{\left(F\right)}=w_{j}^{\left(A\right)}*\left(1-e_{j}\right),\;j=1,2,\ldots n$$

where $w_{j}^{\left(F\right)}$ represents the fine-tuned weight value of index *C_j_*, $w_{j}^{\left(A\right)}$ denotes the weight of index *C_j_* obtained from the AHP method, *e_j_* is the entropy value of index *C_j_*.

Finally, a standardization process can be used to determine the final weight (*w_j_*) of index *C_j_*, as presented in Equation (2).

(2) $$w_{j}=w_{j}^{\left(F\right)}/\sum_{j=1}^{n}w_{j}^{\left(F\right)}$$

#### 2.3.2. GIS Technology

GIS technology can be used to create interactive queries, analyze spatial information, edit data in maps, and present spatial or geographic results of all these operations [57,58,59]. During the process of using GIS, data can be constantly updated in real time, which is in response to rapid changes in the real world. There are a lot of spatial processing analysis tools which can be used to analyze and solve complex problems. Thus, GIS is a commonly used tool in landfill site selection studies. Four popular functions are introduced as follows:

(1) Data extraction. Data extraction is an important function of GIS technology. It can be used to crop, segment, and screen given data, so as to extract required data for further analysis. Thus, it is the prerequisite for utilizing other GIS modeling functions.
(2) Buffer analysis. A buffer is an area whose boundary area consists of a set of defined points at a specified maximum distance, and the size of the area is determined by the radius of the neighborhood or the condition of the buffer set [60]. In the selection of landfill sites, it is necessary to implement buffer analysis to meet local environmental, city planning, and other requirements.
(3) Surface analysis. Surface analysis is obtaining the spatial characteristic information data implied in the existing data, such as slope direction, contour, fill excavation, mountain shadow, slope, curvature, and visibility analysis, etc. Surface analysis is mainly based on raster tools to analyze geological information data.
(4) Overlay analysis. Overlay analysis is an important analysis tool for the establishment of the site selection model and for extracting the implicit space information. Based on the same reference coordinate system, overlay analysis can aggregate and deal with two or more groups of different data to generate new data and new attribute features.

In this study, the geographic data (e.g., surface water, airport information) were collected from governmental departments in Shenzhen, such as the Planning and Land Commission, the Shenzhen terrain platform, and the Huaxia region, etc. Arc GIS Desktop 10.3 was used for data analysis.

## 3. Results

### 3.1. Determination of Weights

In this study, the data used for the AHP-entropy approach were collected from a questionnaire survey. Five experts who had rich knowledge and experience on construction landfill site selection were invited to participate in the survey. The AHP method was implemented using YAAHP software. Before calculating the weights, the consistency ratios of all the judgement matrixes were tested and the results were less than 0.10. After deriving the preliminary weights, the entropy method was then employed to fine tune the weights using MATLAB. The AHP-entropy analysis results of all the influencing factors are shown in Table 3.

Based on the AHP-entropy analysis results, the overall weights can be derived, as shown in Table 4. The overall weights can lay a foundation for establishing a suitable model for construction landfill selection.

### 3.2. Model Establishment

#### 3.2.1. Environmental Criteria

##### Distance to Surface Water

**Figure 1:**
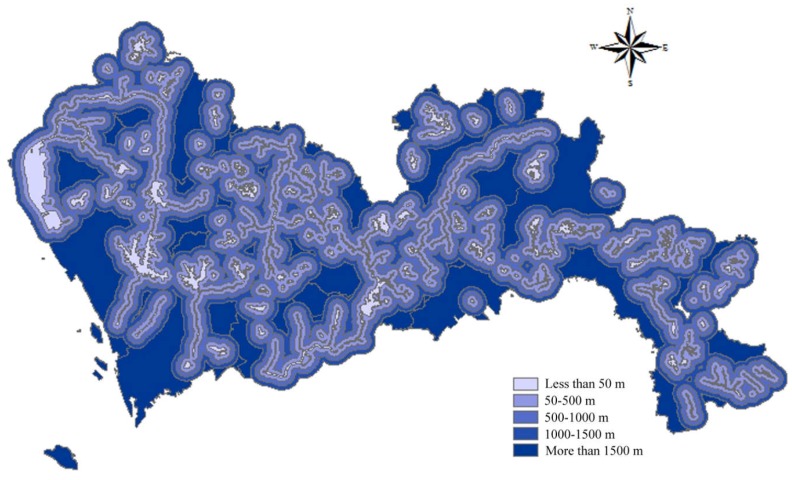
Map of distance to surface water.

According to the Construction Standard for Municipal Solid Waste Landfills, landfills should be located far away from surface water and should be built in the downstream area if possible. More specifically, the Technical Code for Sanitary Landfilling of Domestic Waste stipulates that the distance between landfills and surface water should be larger than 50 m. Furthermore, according to China’s Solid Waste Management Law, the distance between the construction waste landfills and surface water shall not be less than 500 m. Based on the collected requirements, buffer zones were built around the surface water and scores were obtained according to the buffer distance. In this study, a buffer distance less than 50 m obtained 1 point; 50–500 m, 2 points; 500–1000 m, 3 points; 1000–1500 m, 4 points; and a distance more than 1500 m obtained 5 points. The spatial results of the distance to surface water are presented in Figure 1.

##### Distance to Water Source Protection Areas

**Figure 2:**
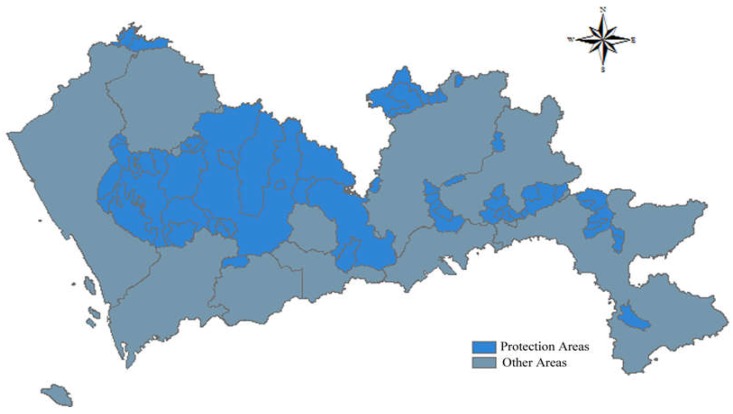
Map of distance to water source protection areas.

Water source protection areas are the specially protected areas established by the government to protect water resources. The Law of the People’s Republic of China on the Prevention and Control of Water Pollution stipulates that water source protection areas include drinking water sources, scenic water, water for important fisheries, and other water sources with special economic and cultural purposes. To protect water sources, the Shenzhen government has promulgated a series of policies, such as the Regulations of Drinking Water Source Protection Area Management in Shenzhen and the Statutory Plan of Shenzhen. Currently, there are 27 water source protection areas in Shenzhen. According to the Pollution Control Standards for Landfills, it is stipulated that landfill must not be in water source protection areas. Thus, the water source protection areas are rated 1 and other areas are ranked 5, as shown in Figure 2.

##### Distance to Nature Reserve

**Figure 3:**
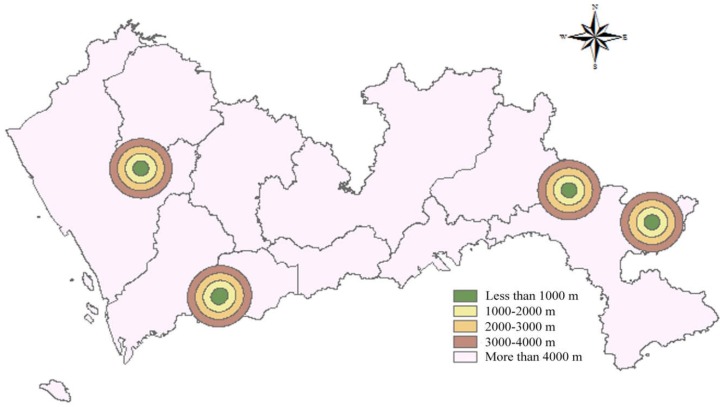
Map of distance to nature reserve.

Nature reserves refer to the natural ecosystems and precious endangered animals and plants and other protected species in the area. There is a total of five nature reserves in Shenzhen. According to the Design Specifications of Landfills and Technical Code for Sanitary Landfilling of Domestic Waste, landfills should not be established in nature reserves and should be more than 1000 m away from the nature reserves. Therefore, buffer zones were set up at 1000 m around the nature reserves and scores were allocated according to the buffer distance. A buffer distance less than 1000 m was allocated 1 point; 1000–2000 m, 2 points; 2000–3000 m, 3 points; 3000–4000 m, 4 points; and a distance more than 4000 m obtained 5 points. The spatial results of the distance to nature reserves are presented in Figure 3.

##### Distance to Airport

**Figure 4:**
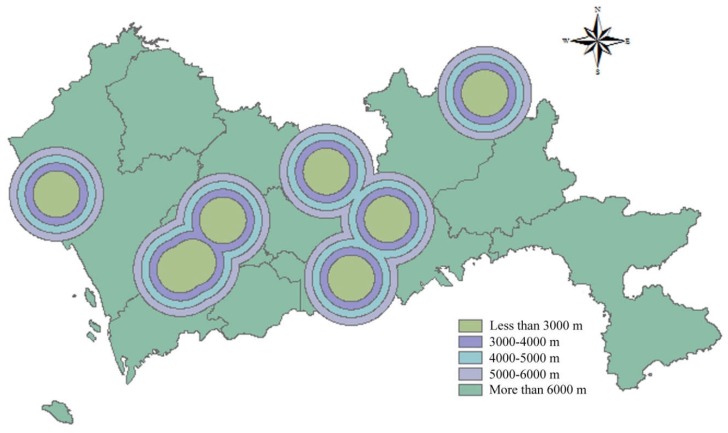
Map of distance to airport.

The Technical Code for Sanitary Landfilling of Domestic Waste stipulates that the distance from the airport to the landfill should be more than 3000 m. Distance between landfills and airports should be as far as possible, the score gradually increases with the increase of buffer distance. In this study, a buffer distance less than 3000 m derived 1 point, the ranges of 3000–4000 m, 4000–5000 m and 5000–6000 m were allocated 2 points, 3 points, and 4 points respectively. For a distance more than 6000 m, 5 points were given. Thus, the spatial results of distance to airport are represented in Figure 4.

##### Special Land and Agricultural Land

**Figure 5:**
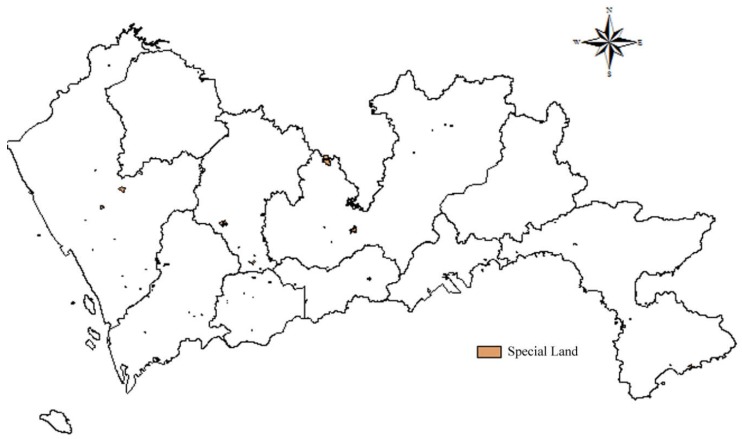
Map of distance to special land.

**Figure 6:**
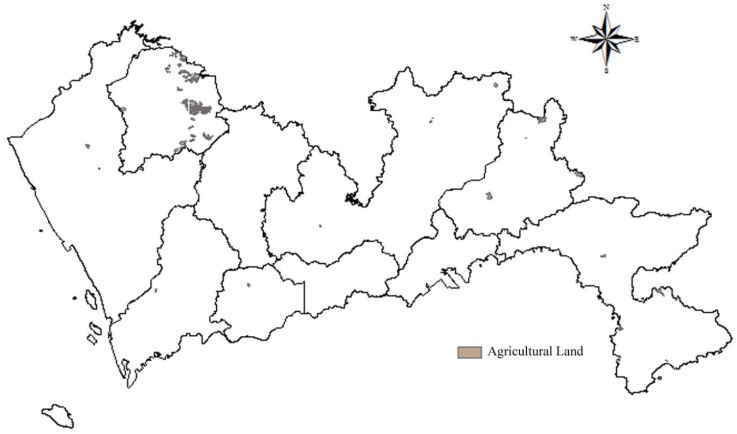
Map of distance to agricultural land.

Special land includes military facilities, embassy sites, religious sites, teaching sites, tombs, etc. Agriculture land includes cultivated land, gardens, woodlands, pastures, and other agricultural productive building land. Special land and agricultural land are usually small in size, few in number, and scattered in the suburbs. The data of special land and agricultural land in Shenzhen are obtained through data screening. According to the Construction Standard for Municipal Solid Waste Landfills, landfills should not be constructed in the agricultural protection zone, worker-peasant planning zone, and other special protection zones. Thus, special land and agricultural land were allocated 1 point, and the spatial results of special land and agricultural land are shown in Figure 5 and Figure 6 respectively.

##### Slope and Altitude

**Figure 7:**
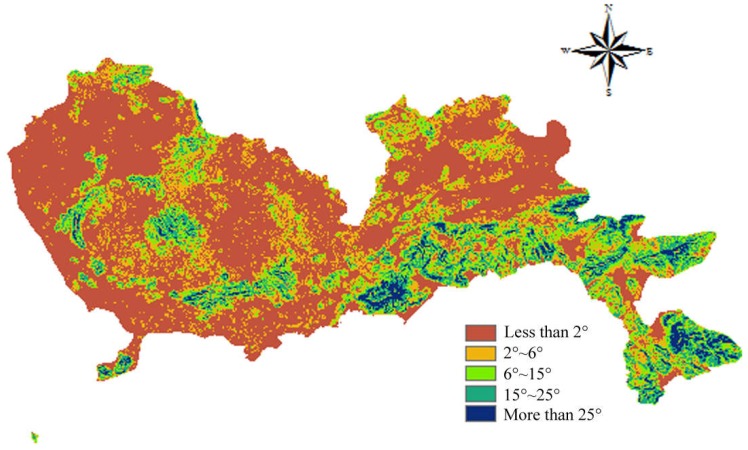
Map of slope.

**Figure 8:**
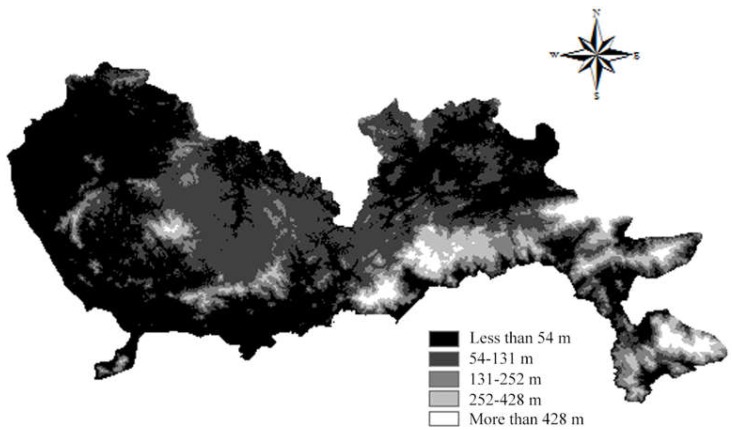
Map of altitude.

According to the Technical Specifications for Construction Waste Disposal and the Construction Standard for Municipal Solid Waste Landfills, the selection of construction waste landfill sites needs to take topography into consideration. In the Technical Regulations for National Land Use Status Survey, the slope of cultivated land is divided into less than 2°, 2°–6°, 6°–15°, 15°–25°, and more than 25°. A smaller slope is regarded as having a lower construction cost. Similarly, a lower altitude is regarded as having a lower construction cost. Therefore, the areas with a slope more than 25° were ranked 1 point while the areas with a slope less than 2° were allocated 5 points. The spatial results of slope are depicted in Figure 7. A similar scoring process was applied to altitude, and the corresponding results are shown in Figure 8.

#### 3.2.2. Social Criteria

##### Distance to Tourist Attractions

**Figure 9:**
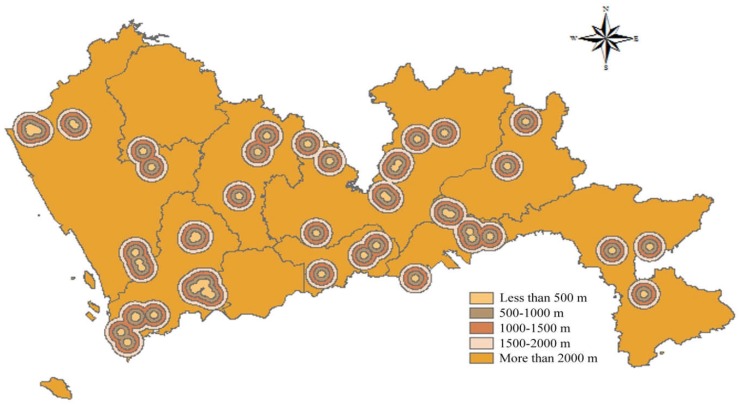
Map of distance to cultural attractions.

**Figure 10:**
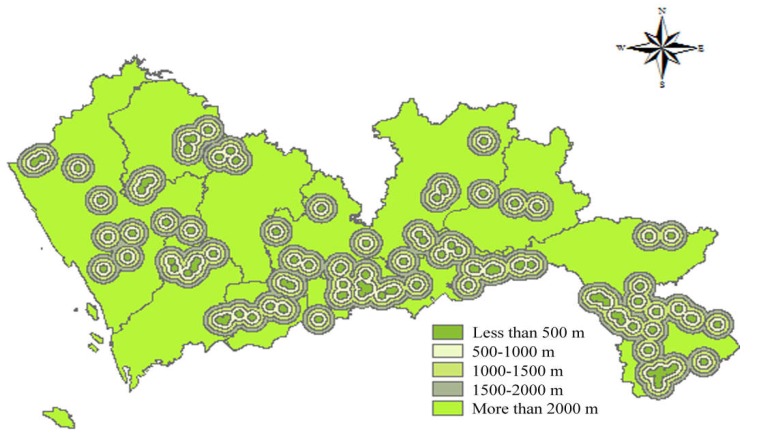
Map of distance to natural attractions.

**Figure 11:**
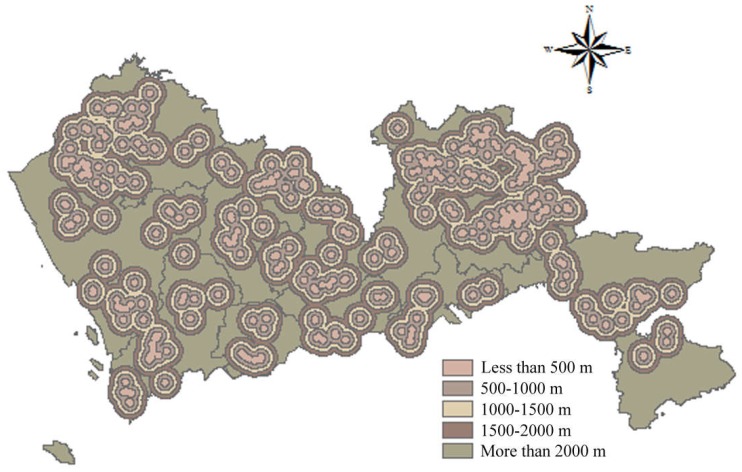
Map of distance to historical relics.

According to the Technical Specifications for Construction Waste Disposal, the Construction Standard for Municipal Solid Waste Landfills, and the Technical Code for Sanitary Landfilling of Domestic Waste, construction waste landfills have a certain impact on the urban environment and landscape. There should be a certain protection distance between landfills and scenic areas, tourist areas, and cultural relics. Thus, all scenic spots, such as cultural attractions, natural attractions and historical relics, should be as far as possible from the landfills. In this study, the buffer with a distance less than 500 m received a score of 1; the buffer with a distance of 500–100 m, a score of 2; the buffer with a distance of 1000–1500 m, a score of 3; the buffer with a distance of 1500–2000 m, a score of 4; and the buffer with a distance greater than 2000 m received a score of 5. The spatial results of cultural attractions, natural attractions, and historical relics are shown in Figure 9, Figure 10 and Figure 11 respectively.

##### Distance to Residents

**Figure 12:**
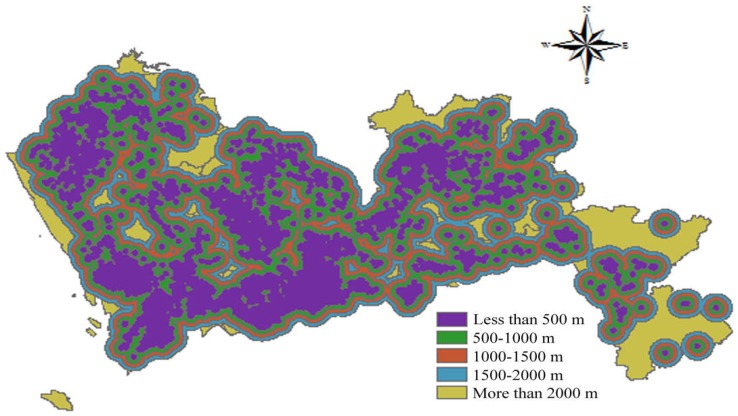
Map of distance to urban settlements.

**Figure 13:**
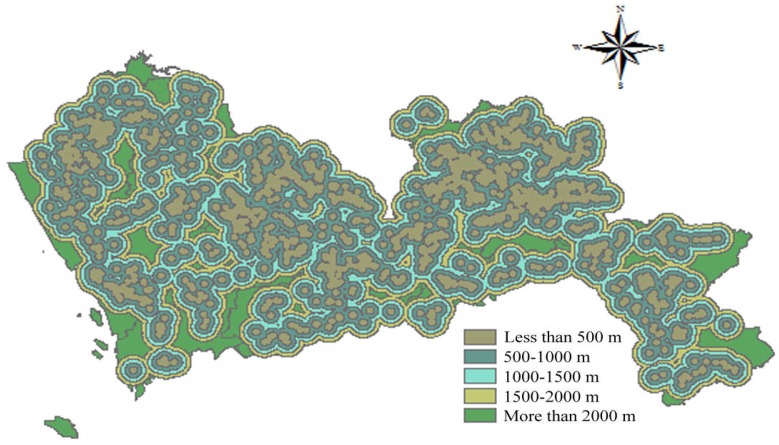
Map of distance to rural settlements.

According to the Construction Standard for Municipal Solid Waste Landfills and the Technical Code for Sanitary Landfilling of Domestic Waste, the impacts of landfills on residents and the surrounding environment should be considered. It is stipulated that the distance from landfills to residential sites should be greater than 500 m. Therefore, buffers were set around urban settlements and rural settlements. A buffer with a distance less than 500 m was allocated a score of 1; a buffer with a distance of 500–1000 m, a score of 2; a buffer with a distance of 1000–1500 m, a score of 3; a buffer with a distance of 1500–2000 m, a score of 4; and a buffer with a distance greater than 2000 m received a score of 5. The spatial results of urban settlements and rural settlements are shown in Figure 12 and Figure 13 respectively.

#### 3.2.3. Economic Criteria

##### Distance to Main Road

**Figure 14:**
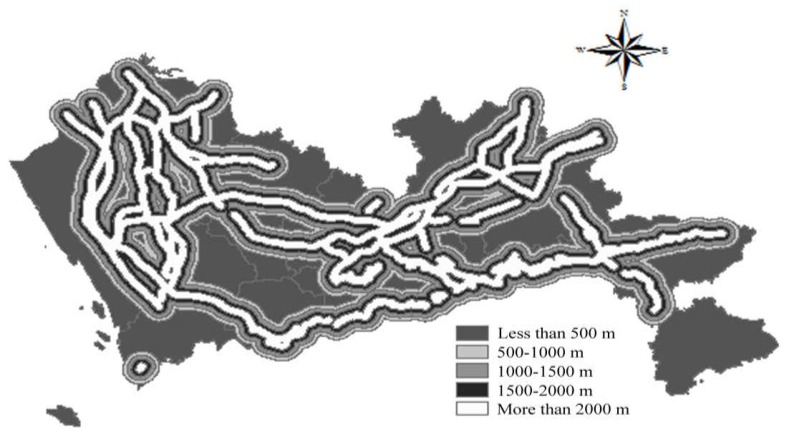
Map of distance to main road.

According to the Construction Standard for Municipal Solid Waste Landfills, Pollution Control Standards for Landfills, and Technical Code for Sanitary Landfilling of Domestic Waste, landfills should locate in areas with convenient transportation and reasonable distances and should have a reasonable positional relationship with trunk roads, highways, railways, etc. A shorter transportation distance can lead to lower transportation cost. The main roads in Shenzhen were screened from Shenzhen Road Network data. A buffer with a distance less than 500 m obtained a score of 1; a buffer with a distance of 500–1000 m, a score of 2; a buffer with a distance of 1000–1500 m, a score of 3; a buffer with a distance of 1500–2000 m, a score of 4; and a buffer with a distance of more than 2000 m was given a score of 5. The spatial results of distance to main road are shown in Figure 14.

##### Distance to Potential Demolished Buildings in the Next 20 Years

**Figure 15:**
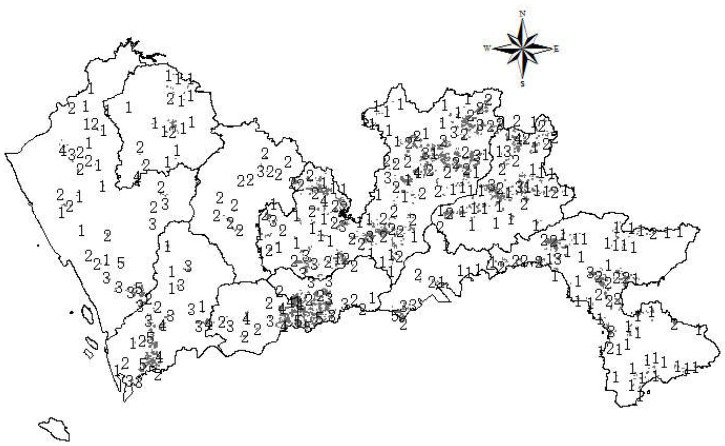
Map of distance to potential demolished buildings in the next 20 years.

In general, the transportation cost will be reduced if landfills can be located closer to the waste generation sites. In terms of construction waste, it is essential to predict the waste generation amount as construction activities are conducted in different locations and at different times. In China, along with the fast development of urban renewal, the amount of waste generated from demolition activities is dramatically larger than waste produced in construction activities [61,62,63]. Thus, this paper predicts the potential demolished buildings in the next 20 years to determine the site selection of construction waste landfills. First, the information of current buildings in Shenzhen was imported into GIS. Assuming a building life is 50 years, then the buildings that have a building life of more than 50 years from 2017 to 2037 were screened from the database. According to the regulations introduced in the above sections, natural and historical heritage, memorial buildings, ancient buildings, and cultural relic protection buildings could not be demolished. Thus, they were excluded from the estimation. According to the Technical Code for Construction Waste Reduction of Shenzhen, the waste generation rates for different types of buildings were derived and the total waste generation was calculated using construction floor areas multiplied by waste generation rates. In this study, a buffer with the weight of 10–1751 tons was allocated a score of 1; a buffer with a weight of 1751–5879 tons, a score of 2; a buffer with a weight ranging from 5879 tons to 15,220 tons, a score of 3; a buffer with a weight of 15,220–38,722 tons, a score of 4; and the buffer with a weight of 38,722–99,908 tons received a score of 5. The spatial results of potential generated waste in the next 20 years are shown in Figure 15.

##### Land Price

**Figure 16:**
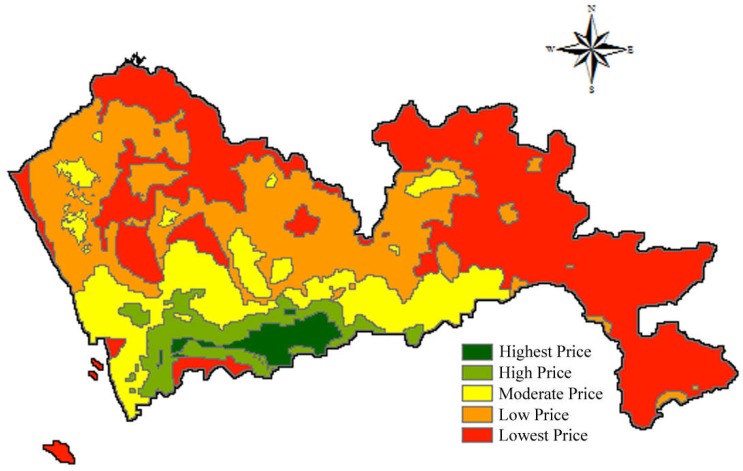
Map of land price.

Land price can influence the selection of landfill sites. Normally, the land price for establishing landfills should be low to reduce construction cost. According to the Shenzhen City Planning and Land Resources Committee, the latest land prices are divided into five categories. Therefore, the land with the highest price received a score of 1 and the land with the lowest price, a score of 5. The spatial results of land price are shown in Figure 16.

### 3.3. Suitability Analysis

With the aid of GIS, an overlay analysis was conducted to integrate all the layer maps considering their weights. Based on the AHP-entropy approach, a weight was appointed to each sub-criterion. The most important factor in site selection is the distance from urban residents, with a weight of 0.3124, followed by the distance from the water conservation area and the distance from the nature reserve, with weights of 0.1119 and 0.0920 respectively. Among the economic factors, this study considered the distribution of construction waste generation in the next 20 years. The weight accounts for 0.0341 in all site selection factors, which is also important for the site selection of construction waste landfills.

**Figure 17:**
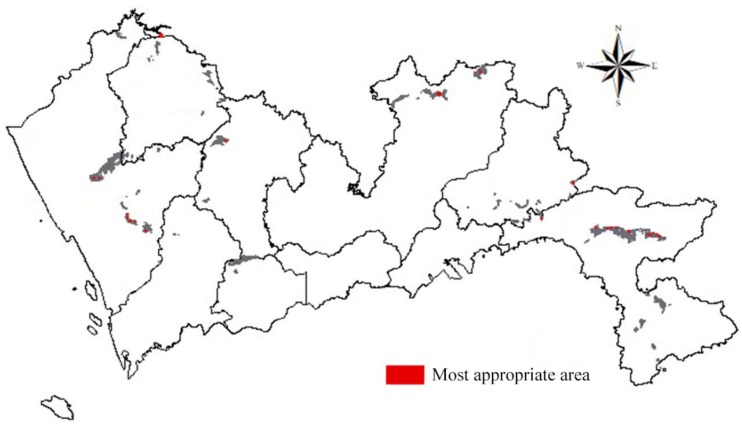
Suitability map of construction waste landfills.

After the overlay analysis, the most appropriate areas for establishing construction landfills were derived, as shown in Figure 17. According to Figure 17, the most suitable area is 7,652,163 m^2^, accounting for 0.38% of the total area. The area that is suitable for the construction waste landfills is 351,123,966 m^2^, accounting for 17.58% of the total area. From Figure 17, it can be seen that the distributions of the most suitable area and the suitable area are very scattered.

## 4. Discussion

According to the analysis results, it can be found that suitable sites for establishing landfills are very limited in Shenzhen. However, along with the fast economic development, the generation of construction waste is expected to be large. Actions must be adopted by the government to stimulate construction stakeholders’ waste reduction, reuse, and recycling behavior. According to the research findings provided by Wu, et al. [64], the most effective strategy to motivate construction practitioners’ waste management behavior is increasing the economic benefits. Thus, it is proposed that the government increase the landfilling fee for construction waste. Moreover, it is recommended that it should be encouraged to establish construction waste recycling facilities. Construction waste should be sent to recycling facilities other than landfills.

Though this research was carefully designed, there are some limitations. For example, there are still some other factors that can affect the site selection of construction waste landfills, such as lithology, groundwater, and permeability. However, these factors were not included in this study because the source data were not achievable. Future research in other regions should be conducted considering these factors.

## 5. Conclusions

In recent years, with rapid economy development and intensive urban renewal, the generation of construction waste has been enormous in many cities in China. At present, the recycling rate of construction waste is only about 5%, and the remaining construction waste is mainly disposed of at landfills. However, many cities in China are facing a problem of running out of existing landfills. Thus, it is essential to set up an appropriate model to determine suitable sites for construction waste landfill.

To develop a reasonable model for selecting suitable sites for establishing construction waste landfills, this study took Shenzhen as an example. The influencing factors were identified, and the AHP-entropy approach was employed to calculate their weights. With the aid of GIS, a suitability model was established for site selection of construction waste landfills. It was calculated that the most suitable landfill area in Shenzhen is 7,652,163 m^2^, accounting for 0.38% of the total area, while a suitable landfill area is 351,123,966 m^2^, accounting for 17.58% of the total area. The remaining area is not suitable for the construction of landfill and this area is 1,638,074,575 m^2^, accounting for 82.04% of the total area. Through the analysis, it was found that the most suitable and suitable areas for establishing landfills are small. Therefore, it is necessary to increase the recycling rate of construction waste and reduce landfills so as to save land resources and protect the environment.

## Figures and Tables

**Table 1:** Summary of existing literature.

Reference	Waste Type	Method Used
Giannikos [14]	Hazardous waste	Multi-objective modeling.
Cheng, et al. [17]	Solid waste	Integration of Multi-Criteria Decision Analysis (MCDA) and Inexact Mixed Integer Linear Programming (IMILP) methods.
Rakas, Teodorović and Kim [15]	General waste	Multi-objective modeling.
Calvo, et al. [18]	Municipal waste	Environmental diagnosis methodology.
Al-Jarrah and Abu-Qdais [19]	Municipal solid waste	Intelligent system based on fuzzy inference.
Eiselt [20]	Municipal solid waste	Mixed-integer linear programming.
Şener, Süzen and Doyuran [16]	General waste	Integration of Geographic Information Systems (GIS) and Multi-Criteria Decision Analysis (MCDA).
Alumur and Kara [21]	Hazardous waste	Multi-objective location-routing model.
Sumathi, et al. [22] Wang, et al. [23]	Solid waste	Multi-Criteria Decision Analysis (MCDA) and overlay analysis using Geographic Information System (GIS).
Şener, et al. [24]	Solid waste	Analytical Hierarchy Process (AHP) and Geographic Information System (GIS).
Şener, et al. [25]	Municipal solid waste	Analytical Hierarchy Process (AHP) and Geographic Information System (GIS).
Eskandari, et al. [26]	Solid waste	Analytical Hierarchy Process (AHP), Geographic Information System (GIS), and remote sensing methods.
Gorsevski, et al. [27]	Municipal solid waste	Analytical Hierarchy Process (AHP) and Geographic Information System (GIS).
Vasiljević, et al. [28]	General waste	GIS-based multi-criteria decision analysis approach.
Abd-El Monsef [29] El Baba, et al. [30]	General waste	Analytical Hierarchy Process (AHP) and Geographic Information System (GIS).
Kharat, et al. [31]	General waste	Analytical Hierarchy Process (AHP), Geographic Information System (GIS), and remote sensing methods.
Torabi-Kaveh, et al. [32]	Solid waste	Multi-Criteria Decision Analysis (MCDA) and Analytical Hierarchy Process (AHP) method.
Rahmat, et al. [33]	Municipal solid waste	Fuzzy-Analytical Hierarchy Process (FAHP) and Technique for Order Preference by Similarity to Ideal Solution (TOPSIS)-based methodology.
Giannikos [14]	Solid waste	Multi-Criteria Decision Analysis (MCDA), Geographic Information System (GIS), and Fuzzy Analytical Hierarchy Process (FAHP).
Cheng, Chan and Huang [17]	Solid waste	A combination of Geographic Information System (GIS) and Analytic Hierarchy Process (AHP).
Guler and Yomralioglu [34]	Solid waste	Analytical Hierarchy Process (AHP) and Geographic Information System (GIS).
Habibi, et al. [35]	Municipal solid waste	A multi-objective robust optimization model.
Hanine, et al. [36]	Industrial waste	An OLAP/GIS-Fuzzy AHP–TOPSIS based methodology.
Krishna, et al. [37]	Solid waste	A geospatial multicriteria approach.
Islam, et al. [38]	Solid waste	Analytic Hierarchy Process (AHP).
Khodaparast, et al. [39]	Municipal solid waste	A combination of Geographic Information System (GIS) and Analytic Hierarchy Process (AHP).
Liu, et al. [40]	Food waste	A hybrid modified MADM model.
Santhosh and Babu [41]	Municipal solid waste	DRASTIC method and Analytical Hierarchy Process (AHP) and Geographic Information System (GIS).
Spigolon, et al. [42]	Municipal solid waste	Multiple decision analysis and geographic information system (GIS) analysis.
Wang, et al. [43]	Municipal solid waste	A fuzzy age-sectioned groundwater environmental health risk assessment model.

**Table 2:** Factors for construction waste landfill site selection.

Criteria	Factor	Sub-Factor
Environmental Criteria (B1)	Distance to surface water (C1)	
	Distance to water source protection area (C2)	
	Distance to nature reserve (C3)	
	Distance to airport (C4)	
	Special land (C5)	
	Agricultural land (C6)	
	Geomorphic topography (C7)	Slope (D1)
		Altitude (D2)
Social Criteria (B2)	Distance to tourist attractions (C8)	Distance to cultural attractions (D3)
		Distance to natural attractions (D4)
		Distance to historical relics (D5)
	Impact on residents (C9)	Distance to urban residents (D6)
		Distance to rural residents (D7)
Economic Criteria (B3)	Transportation cost (C10)	Distance to main road (D8)
		Distance to potential demolished buildings in the next 20 years (D9)
	Land price (C11)	

**Table 3:** AHP-entropy analysis results of all influencing factors.

		AHP Weights	Entropy	Difference Coefficient	Entropy Method Weight	Comprehensive Weight
Criteria	B1	0.5373	0.9741	0.0259	0.303	0.4947
	B2	0.3776	0.9687	0.0313	0.3659	0.4198
	B3	0.085	0.9716	0.0284	0.3311	0.0855
	Sum	0.9999	2.9144	0.0856	1	1
Environmental Criteria	C1	0.1303	0.9862	0.0138	0.1571	0.1671
	C2	0.3564	0.9932	0.0068	0.0777	0.2262
	C3	0.2044	0.9902	0.0098	0.1114	0.1859
	C4	0.0574	0.9859	0.0141	0.1615	0.0757
	C5	0.0839	0.9852	0.0148	0.1688	0.1157
	C6	0.1269	0.9848	0.0152	0.1731	0.1794
	C7	0.0407	0.9868	0.0132	0.1504	0.05
	Sum	1	6.9123	0.0877	1	1
Social Criteria	C8	0.1476	0.9524	0.0476	0.4809	0.1383
	C9	0.8524	0.9486	0.0514	0.5191	0.8617
	Sum	1	1.901	0.099	1	1
Economic Criteria	C10	0.4901	0.9999	0.0001	0.4996	0.4897
	C11	0.5099	0.9999	0.0001	0.5004	0.5103
	Sum	1	1.9998	0.0002	1	1
Geomorphic topography (C7)	D1	0.4785	0.9809	0.0191	0.4311	0.4101
	D2	0.5215	0.9747	0.0253	0.5689	0.5899
	Sum	1	1.9556	0.0444	1	1
Distance from the tourist attractions (C8)	D3	0.2391	0.9928	0.0072	0.2381	0.1359
	D4	0.1891	0.9942	0.0058	0.1924	0.0869
	D5	0.5718	0.9829	0.0171	0.5694	0.7772
	Sum	1	2.9699	0.0301	0.9999	1
Impact on Residents (C9)	D6	0.5597	0.9957	0.0043	0.8329	0.8637
	D7	0.4403	0.9991	0.0009	0.1671	0.1363
	Sum	1	1.9948	0.0052	1	1
Transportation cost (C10)	D8	0.2018	0.9636	0.0364	0.4747	0.186
	D9	0.7982	0.9597	0.0403	0.5253	0.814
	Sum	1	1.9233	0.0767	1	1

**Table 4:** Overall weights of the influencing factors.

Code	Influencing Factors	Weights
C1	Distance to surface water	0.0827
C2	Distance to water source protection area	0.1119
C3	Distance to nature reserve	0.092
C4	Distance to airport	0.0374
C5	Special land	0.0572
C6	Agricultural land	0.0887
D1	Slope	0.0101
D2	Altitude	0.0146
D3	Distance to cultural attractions	0.0079
D4	Distance to natural attractions	0.005
D5	Distance to historical relics	0.0451
D6	Distance to urban residents	0.3124
D7	Distance to rural residents	0.0493
D8	Distance to main road	0.0078
D9	Distance to demolished buildings in the next 20 years	0.0341
C11	Land price	0.0436
